# PD-L1 Exon 3 Is a Hidden Switch of Its Expression and Function in Oral Cancer Cells

**DOI:** 10.3390/ijms24098193

**Published:** 2023-05-03

**Authors:** Lingyan Yan, Yanan Sun, Jihua Guo, Rong Jia

**Affiliations:** 1 The State Key Laboratory Breeding Base of Basic Science of Stomatology (Hubei-MOST) & Key Laboratory of Oral Biomedicine Ministry of Education, School & Hospital of Stomatology, Wuhan University, Wuhan 430079, China; 2 Department of Endodontics, School & Hospital of Stomatology, Wuhan University, Wuhan 430079, China

**Keywords:** programmed cell death 1 ligand 1, exon skipping, antisense oligonucleotide, immunotherapy

## Abstract

The interaction between programmed cell death 1 ligand 1 (PD-L1) and programmed cell death protein 1 (PD-1) protects tumor cells from immune surveillance. PD-L1 exon 3 is a potential alternative exon and encodes an Ig variable (IgV) domain. Here, we found that a lack of exon 3 leads to the significant loss of cellular membrane locations and the dramatically reduced protein expression of PD-L1, indicating that PD-L1 exon 3 is essential for its protein expression and translocation to the cell membrane. Notably, oral cancer cells show almost no exon 3 skipping to ensure the expression of the full-length, functional PD-L1 protein. We discovered two key exonic splicing enhancers (ESEs) for exon 3 inclusion. Two efficient antisense oligonucleotides (ASOs) were identified to block these two ESEs, which can significantly trigger exon 3 skipping and decrease the production of full-length, functional PD-L1 on the surface of cancer cells. Treatment of oral cancer cells with these ASOs significantly enhanced immune cells’ suppression of cancer cell proliferation. Surprisingly, these two ASOs also significantly inhibited cell growth and induced cell pyroptosis in oral cancer cells. Altogether, the results of our study demonstrate the pivotal roles of exon 3 in PD-L1 expression and provide a novel anti-PD-L1 method.

## 1. Introduction

Immunotherapy that targets immune checkpoints, represented by the programmed cell death 1 ligand 1 (PD-L1): programmed cell death protein 1 (PD-1) axis, has revolutionized cancer treatment over recent years. Numerous studies demonstrate that some patients treated with immunotherapy against PD-1/PD-L1 tend to have a favorable prognosis, such as tumor shrinkage, durable responses, and prolonged survival, in a wide range of malignancies, including head and neck cancer [1]. However, two major issues should not be overlooked, namely, the resistance to immunotherapy or the short-lived response to immunotherapy against PD-1/PD-L1 in many patients, the main reason for which is the tumor’s escape from the host’s immune surveillance [2]. Therefore, it is urgent to search for novel therapeutic strategies to enhance the therapeutic effects of immunotherapy against PD-1/PD-L1.

The mechanisms contributing to tumor PD-L1 expression were precise and complex. The interferon gamma (IFN-γ)-induced signaling pathway, Janus kinase (JAK)-signal transducer, and activators of transcription (STAT)–interferon regulatory factor (IRF) 1, can significantly promote PD-L1 expression at the transcriptional level [3]. The activation of the protein kinase B (PKB/AKT)–mammalian target of rapamycin (mTOR) pathway is responsible for PD-L1 protein synthesis, leading to immune escape in lung cancer and glioma [4,5]. In addition, post-translational modifications such as glycosylation [6], phosphorylation [7], ubiquitination [8], acetylation [9], and palmitoylation [10] also participate in the regulation of PD-L1 protein expression and function. However, the regulatory mechanisms of PD-L1 expression and function remain largely unclear.

Alternative splicing (AS) of pre-mRNA is an important molecular event in gene expression and is present in almost 95% of human genes, which greatly enhances the coding capacity of the human genome and enriches proteomic diversity [11]. Abnormal splicing events have been identified to contribute to the development of cancers, such as head and neck squamous cell carcinoma (HNSCC) [12], breast cancer [13], and non-small cell lung cancer [14]. PD-L1 exon 3 encodes the Ig variable (IgV) domain, which is similar to the IgV domain of antibodies as well as T cell receptors, and it serves as a connection between PD-L1 and PD-1 [15]. An exon 3-skipped variant of PD-L1 was detected in activated peripheral blood mononuclear cells (PBMCs) [16]. The deletion of the IgV domain encoded by PD-L1 exon 3 may lead to the disfunction of PD-L1, suggesting a new way for the immunotherapy to target the PD-1/PD-L1 axis.

In this study, PD-L1 exon 3 was identified to be essential for PD-L1 expression and translocation to the tumor cell surface to exercise immune suppressive function. Then, we screened the sequence of human PD-L1 exon 3 and discovered two novel exonic splicing enhancers (ESEs) responsible for its inclusion. Next, we designed and optimized antisense oligonucleotides (ASOs) to block these ESEs, and then, we significantly increased exon 3 skipping and reduced the expression of the functional, full-length PD-L1 on the surface of tumor cells as well as unexpected anti-proliferation and pro-pyroptosis effects in tumor cells.

## 2. Results

### 2.1. The Skipping of PD-L1 Exon 3 Is Rare in Oral Cancer Cells

**Figure 1:**
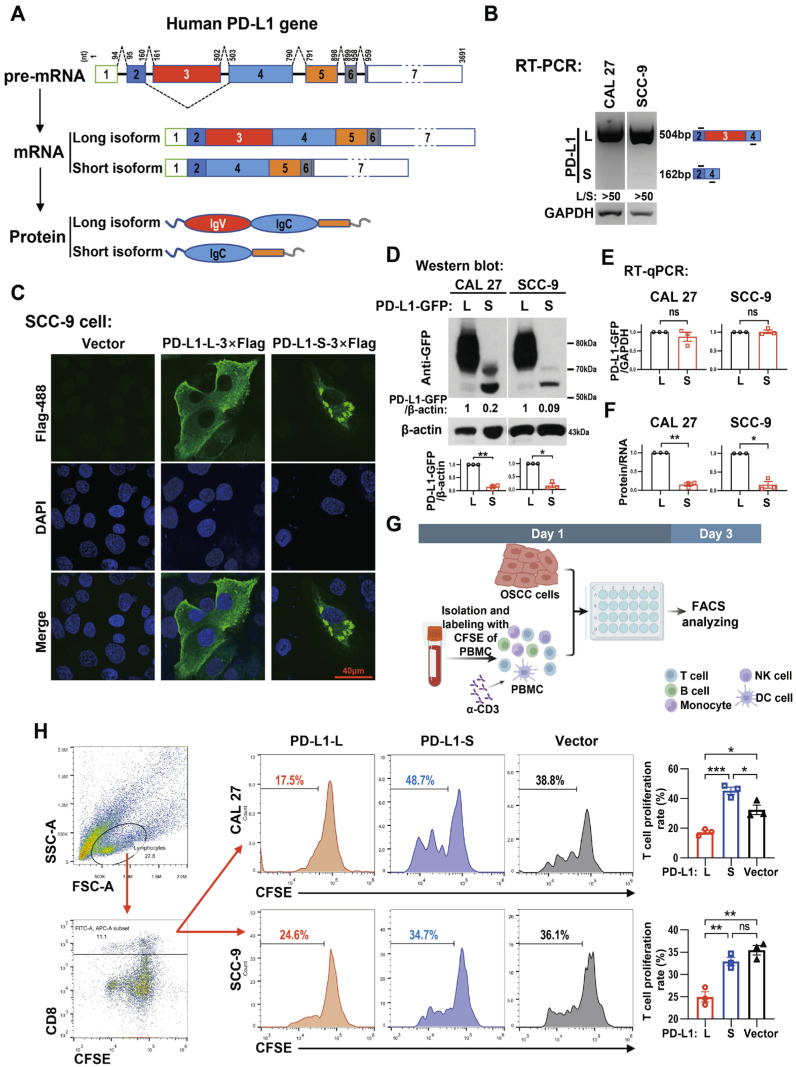
A splice variant of human programmed cell death 1 ligand 1 (PD-L1) mRNA encodes a short protein isoform that lacks the Ig variable (IgV) domain. (**A**) Schematic diagram of the splicing pattern of pre-mRNA and subsequent protein expression pattern of human PD-L1 gene. Human PD-L1 pre-mRNA contains an alternatively spliced exon 3 between exon 2 and exon 4. The numbers above the pre-mRNA exons (boxes) and introns (solid lines) correspond to nucleotide positions in pre-mRNA. Dashed lines above or below introns of pre-mRNA indicate RNA splicing direction. Colored boxes represent the open reading frame (ORF) regions, and colorless boxes represent the untranslated regions (UTR) of PD-L1 pre-mRNA. PD-L1 exon 3 is responsible for encoding the IgV domain of the protein. The full-length PD-L1 mRNA (long isoform) encodes a long isoform protein with IgV domain, whereas the short isoform mRNA with the skipping of exon 3 encodes a short isoform protein lacking the IgV domain. (**B**) The presence of exon 3 skipping in PD-L1 mRNA in oral squamous cell carcinoma (OSCC) cell lines. Reverse transcription PCR (RT-PCR) analysis, which was performed for 37 cycles, detected exon 3 skipping in PD-L1 mRNA in CAL 27 and SCC-9 cells. GAPDH served as a loading control. The schematic diagram on the right shows the spliced products of PD-L1 pre-mRNA. The short lines represent primers. (**C**) Representative fluorescence images of PD-L1 isoform proteins’ expression profiles. SCC-9 cells were transfected with the PD-L1-long isoform-3 × Flag or the PD-L1-short isoform-3 × Flag expression plasmid. The expression of Flag-tagged PD-L1 proteins was analyzed via immunofluorescent assay with rabbit anti-Flag antibody and AlexaFluor^TM^ 488-labeled anti-rabbit IgG antibody after 48 h using a confocal microscope. (**D**–**F**) The expression level of mRNA and protein of green fluorescent protein (GFP)-tagged PD-L1 isoforms in OSCC cell lines. CAL 27 and SCC-9 cells were stably transfected with human PD-L1-long isoform-GFP or PD-L1-short isoform-GFP expression plasmid. Western blot (**D**) and RT-qPCR (**E**) were used to analyze the expression of the exogenous GFP-tagged PD-L1 isoforms, protein, and mRNA levels. β-actin (**D**) and GAPDH (**E**) served as loading controls, respectively. (**F**) Protein/RNA represents the ratio of the relative protein level (normalized by β-actin) to the relative RNA level (normalized by GAPDH). The histograms summarize the ratios of protein to RNA. Data are means ± SEM, n = 3. Student’s *t*-test was used to analyze the differences of the mean values between two groups (PD-L1-L versus PD-L1-S) in (**D**–**F**). (**G**,**H**) Human peripheral blood mononuclear cells (PBMCs) were isolated and labeled with carboxyfluorescein diacetate succinimidyl ester (CFSE), and they were co-cultured with CAL 27 or SCC-9 cells for 3 days with the stimulation of anti-CD3 antibody. Then, PBMCs were stained with anti-CD8 antibody and analyzed with flow cytometry. **(H)** The representative gating strategy for CD8^+^ T cells is shown on the left. The proliferation of CD8^+^ T cells is quantified as the percentages of CFSE-low cells. The histograms on the right summarize the proliferation rate of CD8^+^ T cells. Data are means ± SEM, n = 3. One-way ANOVA followed by Tukey’s test were used to analyze the differences of the mean values among three groups (Vector versus PD-L1-L, Vector versus PD-L1-S, and PD-L1-L versus PD-L1-S). * *p* < 0.05, ** *p* < 0.01, *** *p* < 0.001, ns (not significant).

The human PD-L1 gene contains seven exons (Figure 1A). Among them, exon 3 encodes the IgV domain (Figure 1A), which plays a crucial role in the interaction with its receptor PD-1 [15]. An exon 3-skipped variant of PD-L1 was identified in human PBMCs [16], as is shown in Figure 1A (short isoform). By using a pair of primers located on exon 2 and exon 4, we found that exon 3 skipping truly existed in CAL 27 and SCC-9 oral cancer cells (Figure 1B). However, the expression levels of exon 3-skipped isoform were much lower than full-length exon 3-included isoform (more than 50-fold, Figure 1B), suggesting that cancer cells prefer to include exon 3 and express full-length PD-L1.

### 2.2. Exon 3 Determines the Location of and Is Essential for the Expression and Immunosuppressive Function of PD-L1 Protein

To further explore the intracellular distribution of PD-L1 variants, two different plasmids were constructed, one each for the expression of the full-length long isoform of PD-L1 with exon 3 and the short isoform without exon 3. The long isoform was primarily observed on the surface of cancer cells. However, the short isoform almost exclusively accumulated inside the cells (Figure 1C), where it might lose the ability to interact with PD-1 protein. Surprisingly, the protein expression levels encoded by the short isoform were dramatically lower than those encoded by the long isoform in stably transfected oral cancer cells (Figure 1D), which cannot be attributed to the different transcriptional levels of the two isoforms (Figure 1E,F). These results indicate that exon 3 is essential for not only the localization on the cellular surface, but also the efficient expression of PD-L1 protein.

Next, CAL 27 and SCC-9 cells were co-cultured with carboxyfluorescein diacetate succinimidyl ester (CFSE)-labeled human peripheral blood mononuclear cells (PBMCs), as shown in Figure 1G. The co-culture experiment demonstrates that OSCC cells overexpressing PD-L1-S have a much weaker inhibitory effect on the proliferation of CD8^+^ T cells than PD-L1-L-overexpressed OSCC cells (Figure 1H). These results indicate the crucial roles of PD-L1 exon 3 in the suppression of immune cells by OSCC cells.

### 2.3. Exonic Splicing Enhancers (ESEs) in Exon 3 of the Human PD-L1 Gene

**Figure 2:**
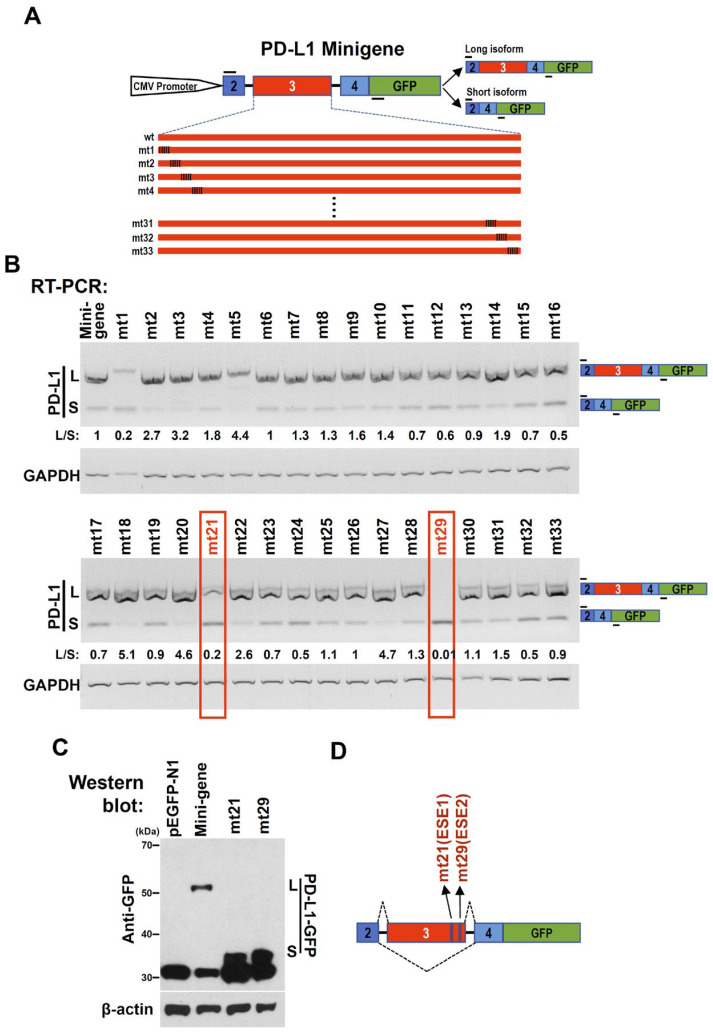
PD-L1 exon 3 contains two exonic splicing enhancers (ESEs). (**A**) Diagram of PD-L1 minigene. Genomic sequence of PD-L1 from exon 2 to 5′ part of exon 4 (without the intermediate sequence of intron 3) was amplified from the genomic DNA of CAL 27 cells and cloned into pEGFP-N1. To map potential regulatory motifs, exon 3 in minigene was serially mutated. (**B**) The PD-L1 minigene or mutated (mt) minigenes were transfected into HEK 293 cells. RT-PCR was used to analyze the alternative splicing of PD-L1 exon 3. The diagram on the right shows the spliced products of PD-L1 minigene. The short line above exon 2 stands for the forward primer, and the short line below the GFP gene stands for the backward primer. L/S represents the ratio of band intensities of long vs. short isoforms. GAPDH served as a loading control. (**C**) Western blot analysis of the protein expression of PD-L1 minigene and mutation (mt21 or mt29) plasmids. β-actin served as a loading control. (**D**) Diagrams of ESE1 (mt21) and ESE2 (mt29).

Given the important roles of exon 3 in the cellular distribution and expression of PD-L1, we focused on the regulatory mechanism of exon 3 alternative splicing. To search the key regulatory elements in PD-L1 exon 3, we constructed a human PD-L1 minigene, including the genomic sequences of PD-L1 from exon 2 to exon 4 (with partial deletion of the long intermediate sequence of intron 3), in which the partial open reading frame of PD-L1 was fused with green fluorescent protein (*GFP*) gene to express a fusion protein (Figure 2A). Then, we performed serial mutations in exon 3 to map potential regulatory elements (Figure 2A). Most of the mutations had no significant effect on the alternative splicing of exon 3. However, it turned out that mutations mt21 and mt29 dramatically promoted exon 3 skipping (Figure 2B). The Western blot results confirm that the wild-type minigene only expresses long PD-L1-GFP fusion protein encoded by long transcript with exon 3, whereas mt21 and mt29 mutation plasmids almost completely switch to express short PD-L1-GFP fusion protein encoded by short transcript without exon 3 (Figure 2C). These results suggest that the corresponding wild-type sequences of mt21 and mt29 contain exonic splicing enhancers (ESEs) for exon 3 inclusion. These two ESEs were named ESE1 and ESE2, respectively (Figure 2D).

### 2.4. Blocking of ESEs to Promote PD-L1 Exon 3 Skipping

**Figure 3:**
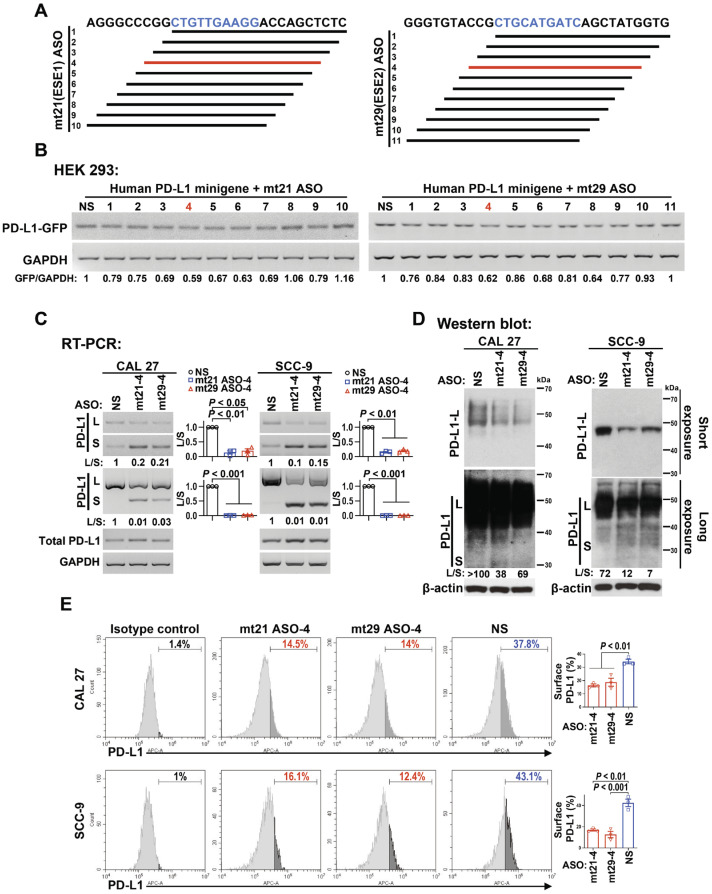
Two antisense oligonucleotides (ASOs), mt21 ASO-4 and mt29 ASO-4, were designed and screened out to block the ESE motifs in PD-L1 exon 3. (**A**) Schematic diagram mapping the ASOs (the line below the sequence) targeting the ESE1 or ESE2 region (highlighted in blue). mt21 ASO-4 and mt29 ASO-4 are highlighted in red. (**B**) HEK 293 cells were co-transfected with PD-L1 minigene and 20 nM ASOs modified with phosphorothioate. The expression of GFP was analyzed via RT-PCR. GAPDH served as a loading control. (**C**) CAL 27 and SCC-9 cells were transfected with 2′-O-methyl-phosphorothioate (2′-O-MOE-PS)-modified ASOs (mt21 ASO-4, mt29 ASO-4, NS). Alternative splicing of PD-L1 exon 3 was detected via RT-PCR using 3 pairs of primers located on exon 3 (forward) and on exon 4 (reverse) for PD-L1-L, the junction of exon 2 and exon 4 (forward) and on exon 4 (reverse) for PD-L1-S, and located on exon 2 (forward) and on exon 4 (reverse) for both PD-L1-L and S. The transcriptional level of PD-L1 was analyzed via RT-PCR using a pair of primers located on exon 5 (forward) and on exon 7 (reverse). GAPDH served as a loading control. Histograms on the right summarize the ratios of band intensities of long vs. short isoforms (L/S). Data are means ± SEM, n = 3. (**D**) Western blot analysis of PD-L1 protein expression (long or short isoform) after transfection with 2′-O-MOE-PS-modified ASOs. β-actin served as a loading control. (**E**) Flow cytometry analysis of cell surface PD-L1 expression. The histograms on the right summarize the rates of OSCC cells expressing surface PD-L1. Data are means ± SEM, n = 3. One-way ANOVA followed by Dunnett’s test were used to analyze the differences of mean values between the control group and the experimental groups (NS versus mt21 ASO-4 and NS versus mt29 ASO-4) in (**C**,**E**).

Next, we tried to promote exon 3 skipping and to inhibit the expression of full-length PD-L1 by blocking these ESEs. Antisense oligonucleotides can basepair target RNA and block specific regulatory elements to control alternative RNA splicing [17]. First, we searched for ASOs that can efficiently bind the ESE1 or ESE2 of PD-L1 exon 3 (Figure 3A). A series of ASOs were modified using phosphorothioate (PS) to enhance their stability in cells. PS-modified ASOs are quite cheap and can promote the cleavage and degradation of the target RNA with RNase H1. Therefore, the more efficiently an ASO binds to the target RNA, the more efficiently the target RNA is degraded. These ASOs were co-transfected with PD-L1 minigene into HEK 293 cells. RT-PCR analysis revealed that mt21 ASO-4 and mt29 ASO-4, which targeted ESE1 and ESE2, respectively, were the most two efficient ASOs for reducing the expression of PD-L1 minigene at the mRNA level (Figure 3B).

We further modified mt21 ASO-4 and mt29 ASO-4 with both PS and 2′-O-methoxyethyl (MOE) modification. 2′-O-MOE modification is expensive, but it allows stronger binding of ASO to the target RNA without inducing RNA degradation, which makes it more appropriate for the regulation of alternative splicing [18]. As expected, 2′-O-MOE-PS-modified mt21 ASO-4 and mt29 ASO-4 remarkably increased the levels of exon 3-skipped short isoforms, whereas they decreased the levels of full-length long isoforms at mRNA levels in both CAL 27 and SCC-9 cells (Figure 3C). No significant changes in the transcriptional level of PD-L1 were observed (Figure 3C).

To further investigate the effect of mt21 ASO-4 and mt29 ASO-4 on PD-L1 protein expression, we performed Western blot analysis using an antibody that targets the C-terminal of the PD-L1 protein. Similar to the almost undetectable mRNA (Figure 1B), the smaller PD-L1 protein encoded by the short isoform was hardly recognized in the Western blot assay in CAL 27 and SCC-9 cells (Figure 3D). Notably, the protein levels of the long isoform were dramatically decreased, whereas those of the short isoform significantly increased, after the treatment of mt21 ASO-4 or mt29 ASO-4 (Figure 3D) in OSCC cells. Meanwhile, in accordance with the essential role of exon 3 in the translocation of PD-L1 protein to the cell surface, we observed that mt21 ASO-4 and mt29 ASO-4 significantly reduced the expression of PD-L1 on the cell surface in CAL 27 and SCC-9 cells, which was observed using flow cytometry (Figure 3E). These results suggest that mt21 ASO-4 and mt29 ASO-4 could efficiently reduce PD-L1 expression on the surface of cancer cells by inducing PD-L1 exon 3 skipping.

### 2.5. mt21 ASO-4 and mt29 ASO-4 Are Effective in Multiple Cancer Cells

**Figure 4:**
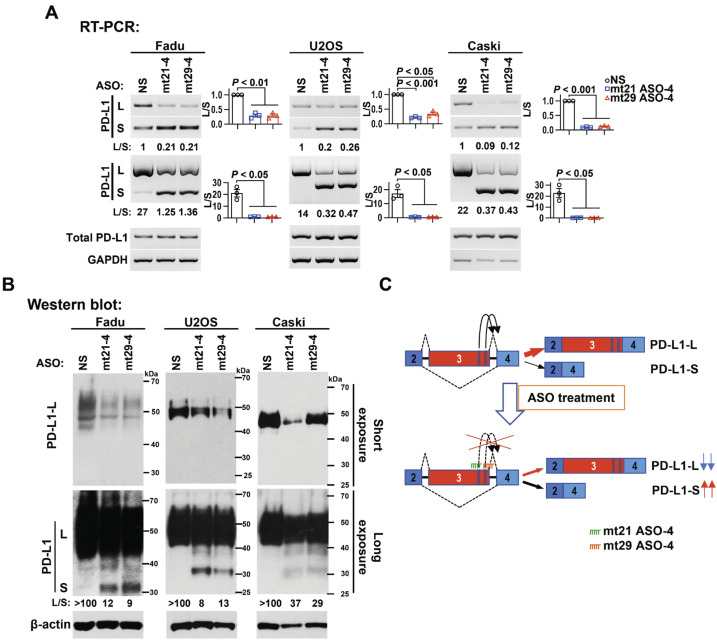
mt21 ASO-4 and mt29 ASO-4 promote PD-L1 exon 3 skipping in several cancer cells. The human hypopharyngeal carcinoma cell line Fadu, human osteosarcoma cell line U2OS, and human cervical carcinoma cell line Caski were transfected with 2′-O-MOE-PS-modified ASOs. (**A**) RT-PCR was used to analyze the alternative splicing of PD-L1 exon 3 and the transcriptional level of PD-L1 mRNA. GAPDH served as a loading control. The histograms on the right summarize the ratios of band intensities of long vs short isoforms (L/S). Data are means ± SEM, n = 3. One-way ANOVA followed by Dunnett’s test were used to analyze the differences of the mean values between the control group and the experimental groups (NS versus mt21 ASO-4 and NS versus mt29 ASO-4). (**B**) A Western blot assay was used to analyze the protein expression of PD-L1 (long or short isoform). β-actin served as a loading control. (**C**) Diagram of PD-L1 exon 3 alternative splicing. The binding and blocking of mt21 ASO-4 and mt29 ASO-4 to ESE1 and ESE2, respectively, promote exon 3 skipping and lead to a decrease in PD-L1 long isoform expression.

Subsequently, we transfected the 2′-O-MOE-PS-modified ASOs into several types of cancer cells, including the human hypopharyngeal carcinoma cell line Fadu, human osteosarcoma cell line U2OS, and human cervical carcinoma cell line Caski, to test the potential “broad spectrum” anti-tumor effect. Likewise, mt21 ASO-4 and mt29 ASO-4 also significantly promoted PD-L1 exon 3 skipping in Fadu, U2OS, and Caski cells at the mRNA level (Figure 4A), and they reduced the protein levels of the full-length long isoform and increased those of the exon 3-skipped short isoform (Figure 4B).

These results demonstrate that the two newly designed ASOs, mt21 ASO-4 and mt29 ASO-4, which target ESE1 and ESE2, respectively, in PD-L1 exon 3, can dramatically promote exon 3 skipping, leading to a significantly decreased expression of full-length functional PD-L1 protein (Figure 4C).

### 2.6. mt21 ASO-4 and mt29 ASO-4 Inhibited Interferon Gamma (IFN-γ)-Induced PD-L1 Expression

**Figure 5:**
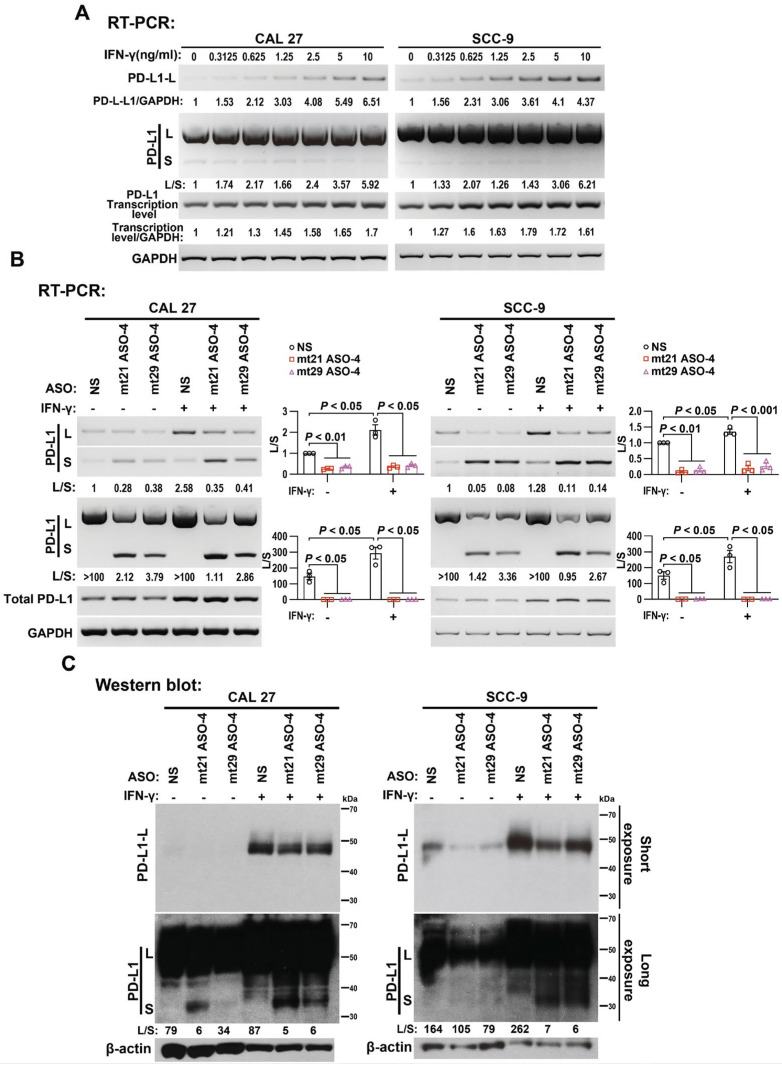
mt21 ASO-4 and mt29 ASO-4 partially reduces interferon gamma (IFN-γ)-induced increases in full-length PD-L1 expression. (**A**) RT-PCR analysis of PD-L1 expression at mRNA level after cells were treated with IFN-γ for 24 h. GAPDH served as a loading control. (**B**,**C**) CAL 27 and SCC-9 cells were pre-treated with IFN-γ or PBS for 12 h. Then, cells were transfected with 2′-O-MOE-PS-modified ASOs mt21 ASO-4, mt29 ASO-4, and NS for 48 h in combination with IFN-γ or PBS treatment. (**B**) The alternative splicing of exon 3 and the transcriptional level of PD-L1 were analyzed via RT-PCR. GAPDH served as a loading control. The histograms on the right summarize the ratios of the band intensities of long vs. short isoforms (L/S). Data are means ± SEM, n = 3. One-way ANOVA followed by Dunnett’s test were used to analyze the differences of the mean values between the control group and the experimental groups (NS versus mt21 ASO-4 and NS versus mt29 ASO-4). (**C**) A Western blot assay was used to analyze the protein expression of the long and short isoforms of PD-L1. β-actin served as a loading control.

The binding of IFN-γ to its receptor on the surface of tumor cells leads to the significant upregulation of PD-L1 through the Janus kinase (JAK)1/JAK2- signal transducer and activators of the transcription (STAT)1/STAT2/STAT3- interferon regulatory factor (IRF) 1 axis [3]. Unexpectedly, we found that the ratios of the long isoform to the short isoform of PD-L1 mRNA significantly increased after IFN-γ treatment in a dose-dependent manner (Figure 5A), which suggests another mechanism that IFN-γ enhances full-length PD-L1 expression.

Although widely identified as an important effector molecule in anti-tumor immunity, IFN-γ may also exert tumor-promoting side effects by enhancing PD-L1 expression [19]. To counteract such side effects, we transfected either mt21 ASO-4 or mt29 ASO-4 into IFN-γ-treated OSCC cells. Consistently, these ASOs were also effective in IFN-γ-treated CAL 27 and SCC-9 cells, showing a significant decrease in long isoforms and a dramatic increase in short isoforms in the mRNA (Figure 5B) and protein (Figure 5C) levels of PD-L1. All of these results indicate that mt21 ASO-4 and mt29 ASO-4 could partially compromise the increased expression of full-length PD-L1 induced by IFN-γ.

### 2.7. mt21 ASO-4 and mt29 ASO-4 Inhibit Cell Growth and Induce Cell Pyroptosis in OSCC

**Figure 6:**
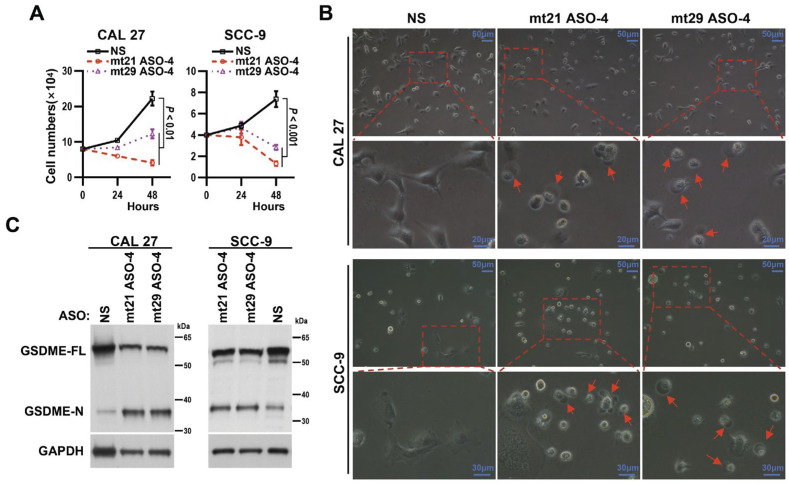
mt21 ASO-4 and mt29 ASO-4 attenuates the proliferation of and induces pyroptosis in OSCC cells. (**A**) Proliferation curves of OSCC cell lines CAL 27 and SCC-9 after transfection with 2′-O-MOE-PS-modified ASOs. Measurements of cell numbers were taken after 24 and 48 h. Data are means ± SEM, n = 4. One-way ANOVA followed by Dunnett’s test were used to analyze the differences of the mean values between the control group and the experimental groups (NS versus mt21 ASO-4 and NS versus mt29 ASO-4). (**B**) Representative images of the morphology of dying OSCC cells after transfection with 2′-O-MOE-PS-modified ASOs. The red arrows indicate cell swelling with large bubbles. (**C**) A Western blot assay was used to analyze the cleavage of gasdermin E (GSDME) after the treatment of ASOs in CAL 27 or SCC-9 cells. GAPDH served as a loading control.

To observe the effects of mt21 ASO-4 and mt29 ASO-4 on the survival of OSCC cells, we performed cell growth assays. Surprisingly, we found that the transfection of 2′-O-MOE-PS-modified mt21 ASO-4 and mt29 ASO-4 induced dramatic decreases in cell proliferation rates in both CAL 27 and SCC-9 cells (Figure 6A). Then, we observed cell swelling and large bubbles, the typical morphology of cells with pyroptosis, in cells transfected with either mt21 ASO-4 or mt29 ASO-4, but not in cells transfected with NS (Figure 6B). Pyroptosis is commonly defined as gasdermin-mediated programmed death, with the cleavage of gasdermin protein as another typical characteristic [20]. In this study, the 2′-O-MOE-PS-modified mt21 ASO-4 and mt29 ASO-4 significantly induced the protein cleavage of gasdermin E (GSDME) in CAL 27 and SCC-9 cells (Figure 6C).

### 2.8. mt21 ASO-4 and mt29 ASO-4 Partially Attenuate the Suppressive Effect of OSCC Cells on Immune Cells

**Figure 7:**
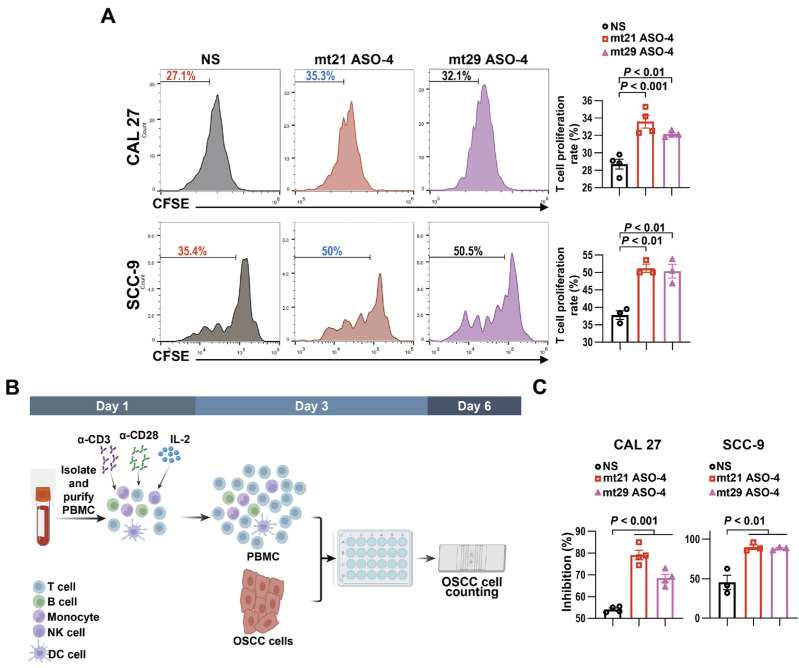
mt21 ASO-4 and mt29 ASO-4 partially overcame the immune suppression induced by OSCC cells. (**A**) Human PBMCs were isolated and labeled with CFSE, and they were co-cultured with CAL 27 or SCC-9 cells transfected with 2′-O-MOE-PS-modified ASOs for 3 days with the stimulation of anti-CD3 antibody. Then, PBMCs were stained with monoclonal antibodies against CD8. Proliferation was quantified as the percentages of CFSE-low cells among CD8^+^ cells. (**B**,**C**) Human PBMCs were isolated and stimulated with anti-CD3/CD28 and IL-2 for 3 days. After being transfected with 2′-O-MOE-PS-modified ASOs, human OSCC cells were either cultured alone or co-cultured with human PBMCs. After 72 h, the suspended PBMCs were washed away, and the adherent live OSCC cells were counted. (**C**) Inhibition was calculated in the following way: (OSCC cells cultured alone—OSCC cells co-cultured with PBMCs)/OSCC cells cultured alone × 100%. Data are means ± SEM, n = 4 or 3. One-way ANOVA followed by Dunnett’s test were used to analyze the differences of the mean values between the control group and the experimental groups (NS versus mt21 ASO-4 and NS versus mt29 ASO-4) in (**A**,**C**).

Considering that the PD-1/PD-L1 interaction represents a pivotal immune-suppressive signaling pathway that facilitates tumor progression, we wondered if a treatment of mt21 ASO-4 or mt29 ASO-4 that targeted tumor cells could enhance the killing effect of immune cells on tumor cells. First, after treatment with 2′-O-MOE-PS-modified mt21 ASO-4 and mt29 ASO-4, CAL 27 and SCC-9 cells were co-cultured with CFSE-labeled PBMCs, as shown in Figure 1E. The inhibition of OSCC cells on the proliferation of PBMCs was significantly attenuated after treatment with mt21 ASO-4 and mt29 ASO-4 (Figure 7A).

Then, we performed a co-culture experiment, as shown in Figure 7B. Notably, mt21 ASO-4 and mt29 ASO-4 not only inhibited the growth of tumor cells, but this inhibition was even more powerful in the presence of immune cells (Figure 7C). Taken together, all of these results indicate that mt21 ASO-4 and mt29 ASO-4 can inhibit the survival of OSCC cells, especially in the immune environment.

## 3. Discussion

A splicing variant of PD-L1 that lacks exon 3 was identified in cancer cells in the present study. However, we found that exon 3 skipping was rare in tumor cells. Importantly, we discovered that exon 3 was essential for the expression of cellular surface full-length PD-L1 protein. Currently, studies of the alternative splicing of PD-L1 mainly reported the extracellular secretory PD-L1 (sec-PD-L1) isoforms, the mRNA isoforms of which lack or partially lack exon 5 that is responsible for encoding the transmembrane domain [21,22]. In addition, the alternative splicing of the 3′ part of PD-L1 exon 3 produces two short, secreted isoforms due to premature stop codons [23]. However, these PD-L1 isoforms still have inhibitory effects on immune cells [23]. In the present study, we demonstrated that exon 3 skipping led to the intracellular distribution of PD-L1 protein and less expression, suggesting a new insight into immune therapy targeting PD-1/PD-L1.

In this study, we uncovered two ESEs required for the inclusion of exon 3 and designed two antisense oligonucleotides (mt21 ASO-4 and mt29 ASO-4) that suppressed the surface PD-L1 expression of tumor cells by blocking these two ESEs and promoting PD-L1 exon 3 skipping. Although monoclonal antibody (mAb)-based immunotherapy, especially using anti-PD-1/PD-L1 antibodies, has achieved great success in the treatment of multiple malignancies, only a minority of patients exhibit dramatic responses to single-agent therapy in clinics [24]. Moreover, the antibodies for tumor treatment still have some disadvantages. First, the large size of antibody makes it difficult to penetrate into the tumor microenvironment, resulting in a significant reduction in efficacy [25]. Then, because PD-1/PD-L1 expression is not limited to the surface of tumor cells and T cells, the indiscriminate neutralization of the antibody may cause undesirable side effects [26]. Meanwhile, although mAb are generally considered to be well tolerated in humans, some components may still be recognized by the recipient as foreign and subsequently lead to a violent immune response, such as acute anaphylactic (IgE-mediated) and anaphylactoid reactions against the mAb, serum sickness, tumor lysis syndrome, and cytokine release syndrome [27]. Compared with anti-PD-L1 antibody, the alternative splicing of PD-L1 exon 3 is a native endogenous regulatory mechanism to control PD-L1 expression. Our results provide a new insight into immunotherapy by modulating the expression of immune checkpoint molecules through adjusting the alternative splicing of their pre-mRNA.

Recently, targeting RNA became a promising therapeutic avenue to combat cancer because of its precision. For example, small molecules targeting RNA have been widely discussed in anti-tumor immune therapy, such as small interfering RNAs (siRNAs) [28,29] or micro RNAs (miRNAs) [30] targeting the PD-1/PD-L1 axis. In addition, antisense oligonucleotide (ASO) is another promising therapeutic agent targeting RNA. The occupancy effects on the target RNA, such as the occupancy of splice sites, make it possible for ASOs to regulate the alternative splicing of pre-mRNA, which has inspired the development of some drugs for the treatment of diseases, such as eteplirsen for Duchenne muscular dystrophy [31] and nusinersen for spinal muscular atrophy [32]. In the field of tumor immunotherapy, researchers have designed some ASOs that silenced immune suppresser gene expression, such as cytotoxic T-lymphocyte associated protein 4 (CTLA-4) and forkhead box P3 (Foxp3), and delayed tumor growth in murine melanoma models [33]. In the current study, after modification with 2′-O-MOE-PS, mt21 ASO-4 and mt29 ASO-4 significantly inhibited the expression of full-length PD-L1 by promoting exon 3 skipping instead of gene silencing, offering an alternative choice for anti-PD-1/PD-L1 therapy.

Moreover, small molecule therapy targeting immune checkpoints has become another promising strategy in cancer immune therapy. For example, the application of a PD-L1 B-cell epitope peptide vaccine (PDL1-Vaxx)-induced polyclonal anti-PD-L1 antibodies and significantly inhibited tumor growth in vivo [34]. Similarly, an anti-PD-1 B-cell epitope vaccine (PD1-Vaxx) also exhibited promising antitumor effects in vivo [35]. In addition, a PD-1-derived small-molecule inhibitor, CA-170, displayed in vivo immune pharmacodynamics and efficacy and inhibited tumor growth [36]. In addition, the small-molecule BMS1166 prevented the transportation of PD-L1 protein from the endoplasmic reticulum (ER) to the Golgi through the regulation of PD-L1 glycosylation and subsequently blocked PD-L1/PD-1 signaling [37]. The ASO targeting metadherin (*MTDH*), a gene can promote PD-L1 expression, was able to improve anti-PD-1 mAb therapy in hepatocellular cancer (HCC) [38]. Moreover, both nucleic acid drugs and small-molecule drugs are receiving increasing attention due to their easy synthesis and slight allergic reactions. In our previous study, the combination of ASO targeting oncogene serine and arginine rich splicing factor 3 (*SRSF3*) with paclitaxel, a small-molecule antitumor drug derived from natural plants, obtained superior inhibition of cell proliferation and the induction of cellular apoptosis compared with monotherapy in OSCC cells [39]. In immune therapy targeting immune checkpoints, the combination of ASOs with small-molecule drugs deserves deeper exploration.

IFN-γ can increase PD-L1 expression, including the expression of both full-length and secreted PD-L1 isoforms [40], through enhancing transcription [3], which strengthens the escape of tumor cells from immune surveillance and affects the response of tumor patients to immune checkpoint therapy [40]. In this study, we discovered that IFN-γ can actually increase the inclusion of PD-L1 exon 3 and the expression of full-length PD-L1 protein. Moreover, the 2′-O-MOE-PS-modified mt21 ASO-4 and mt29 ASO-4 can inhibit the expression of full-length PD-L1 induced by IFN-γ, providing new approaches to compromise the side effects of IFN-γ-related anti-tumor therapy.

## 4. Materials and Methods

### 4.1. Cells and Reagents

Human oral squamous cell carcinoma (OSCC) cell line CAL 27, human hypopharyngeal carcinoma cell line Fadu, human osteosarcoma cell line U2OS, and human embryonic kidney (HEK) 293 cells were cultured in Dulbecco’s modified Eagle medium (DMEM; HyClone, Marlborough, MA, USA) supplemented with 10% fetal bovine serum (FBS; HyClone, Marlborough, MA, USA) and 1% antibiotic–antimycotic (Gibco, Carlsbad, CA, USA). OSCC cell line SCC-9 was cultured in DMEM:F12 (HyClone, Marlborough, MA, USA) supplemented with 10% FBS, 400 ng/mL hydrocortisone, and 1% antibiotic–antimycotic. Human cervical carcinoma cell line Caski was grown in Roswell Park Memorial Institute (RPMI)-1640 medium (HyClone, Marlborough, MA, USA) supplemented with 10% FBS and 1% antibiotic–antimycotic.

CAL 27, SCC-9, and HEK 293 cells were obtained as previously reported [41]. Fadu, U2OS, and Caski cells were purchased from Procell Life Science (Procell, Wuhan, China).

Recombinant human IFN-γ was purchased from Peprotech (#300-02, Peprotech, Rocky Hill, NJ, USA).

### 4.2. Plasmids

For the construction of the open reading frame of PD-L1-S, pCMV3-PD-L1-GFPSpark plasmid (#HG10084, Sino Biological, Beijing, China) was used as a template. First, PCR was conducted with the following pairs of primers: 5′ CCACAGGTGTCCACTCCCAG 3′ and 5′ TTGGTTGATTTTGTTGTATGGGGCGTTCAGCAAATGCCAGTAGG 3′, and 5′ CCTACTGGCATTTGCTGAACGCCCCATACAACAAAATCAACCAA 3′ and 5′ CCACTCAGGACTTGATGGTCACT 3′. The two PCR fragments were ligated in an overlapping PCR and then cloned into pCMV3-PD-L1-GFPSpark plasmid at KpnI and BamHI to replace the fragment containing exon 3.

For the expression of PD-L1 fused with a 3 × Flag tag, the open reading frame was cloned from pCMV3-PD-L1-GFPSpark (#HG10084, Sino Biological, Beijing, China) with the primers 5′ TTCCGGAATTCGCCACCATGAGGATATTTGCTG 3′ and 5′ TTCGCGCGGCCGCCTACTTGTCATCGTCATCCTTGTAGTCGATGTCATGATCTTTATAATCACCGTCATGGTCTTTGTAGTCTTTTGCCGCAGCTTC 3′, and it was cloned into pLVX-IRES-PURO vectors at EcoRI and NotI sites.

The genomic DNA sequences from exon 2 to the 5′ part of intron 3 and from the 3′ part of intron 3 to exon 4 of human PD-L1 were amplified from CAL 27 genome with the following primers: 5′ TTCCGGCTAGCGGCATTCCAGAAAGATGAGGAT 3′ (exon 2) and 5′ GCTCTGTGTTGTTTGTCTCTGGAT 3′ (intron 3), and 5′ TTCCGGAATTCAGGTGTTCCCCACGGCTGA 3′ (intron 3) and 5′ TCCTCTCTCTTGGAATTGGTGGTG 3′ (exon 4). These two fragments were successively cloned into the pEGFP-N1 vector, with exon 2 paired to the 5′ part of intron 3 at NheI and EcoRI sites and the 3′ part of intron 3 paired to exon 4 at EcoRI and BamHI sites, thereby constructing the minigene of human PD-L1. To search the key regulatory elements in PD-L1 exon 3, the sequence of exon 3 in the minigene was serially mutated every 10 bases, which was made by substituting a base with its complementary base at every other base (Figure 2A).

### 4.3. Antisense Oligonucleotides and Transfection

Antisense oligonucleotides (ASOs) modified by phosphorothioate or/and 2′-O-methoxyethyl (MOE)-phosphorothioate were synthesized by Sangon Biotech (Shanghai, China). ASOs were transfected into cells using the Lipofectamine 3000 Reagent (#L3000015, Invitrogen, Carlsbad, CA, USA) according to the manufacturer’s protocol. The sequences of the ASOs were listed in Table S1.

### 4.4. RNA Extraction, Semiquantitative Reverse Transcription PCR (RT-PCR), and Real-Time Quantitative Reverse Transcription PCR (RT-qPCR)

Total RNA was purified using the Total RNA Miniprep Kit (#AP-MN-MS-RNA, Axygen, Union City, CA, USA). Total RNA was treated with DNase I (#M1682, Thermo Scientific, Carlsbad, CA, USA) to remove genomic DNA contamination. Then, reverse transcription was performed using the Maxima H Minus cDNA Synthesis Master Mix (#M1682, Thermo Scientific, Carlsbad, CA, USA) by following the manufacturer’s protocol. Semiquantitative PCR was performed using Green Taq Mix (#P131, Vazyme Biotech, Nanjing, China). Quantitative real-time PCR was performed using Universal SYBR Green Fast qPCR Mix (#RK21203, Abclonal, Wuhan, China). The primers involved in these experiments are listed in Table S2.

### 4.5. Western Blot Analysis

Total cellular protein was collected with 2 × SDS sample buffer and subsequently denatured for 3 min at 95 °C. The protein samples were separated in 10% Nu-PAGE Bis–Tris gel (Invitrogen, Carlsbad, CA, USA) and transferred to nitrocellulose membranes (Pall Corporation, Port Washington, NY, USA). After being blocked with 5% (w/v) milk for 1 h, the membranes were incubated with the following antibodies: rabbit anti-PD-L1 antibody (#28076, 1:2000, Proteintech, Wuhan, China), mouse anti-β-actin antibody (#sc-47778, 1:1000, Santa Cruz Biotechnology, Dallas, TX, USA, USA), rabbit anti-GSDME antibody (#ab215191, 1:2000, Abcam, Cambridge, UK, USA), and mouse anti-GAPDH (#sc-47724, 1:1000, Santa Cruz Biotechnology, Dallas, TX, USA).

### 4.6. Flow Cytometry

CAL 27 and SCC-9 cells were transfected with ASOs and harvested after 48 h. The expression of surface PD-L1 on the cell membrane was detected with flow cytometry using APC-labeled anti-human PD-L1 antibody (#17-5983-42, MIH1, eBioscience, San Diego, CA, USA) in CytoFlex (Beckman, Indianapolis, IN, USA).

### 4.7. Peripheral Blood Mononuclear Cell Isolation, Labeling, and Co-Culture Assay

Peripheral blood mononuclear cells (PBMCs) were isolated from healthy donors.

For the proliferation analysis of PBMCs, PBMCs were labeled with CFSE (5 μM, #65-0850, Thermo Scientific, Carlsbad, CA, USA) and co-cultured with OSCC cells in ImmunoCult™-XF T Cell Expansion Medium (STEMCELL, Vancouver, BC, Canada) supplemented with anti-CD3 antibody (#555336, 1 μg/mL, BD Pharmingen, San Diego, CA, USA) for 3 days. Then, PBMCs were harvested and stained with anti-CD8 cell surface marker antibody (#301014, Biolegend, San Diego, CA, USA) and were analyzed using flow cytometry.

For the proliferation analysis of OSCC cells, PBMCs were cultured in ImmunoCult™-XF T Cell Expansion Medium (STEMCELL, Vancouver, BC, Canada) and were activated with human CD3/CD28 T cell Activator (#10971, 25 uL/mL, STEMCELL, Vancouver, BC, Canada) and IL-2 (#78036, 10 ng/mL, STEMCELL, Vancouver, BC, Canada) for 3 days. Subsequently, OSCC cells transfected with ASOs were co-cultured with activated PBMCs in 24-well plates for 3 days. Then, the suspended PBMCs were washed away, and the adherent live OSCC cells were counted.

### 4.8. Immunofluorescence

For immunofluorescence, 4% paraformaldehyde (PFA, #G1101, Servicebio, Wuhan, China)-fixed cell samples were permeabilized in 0.4% Triton-X100 (#0694, Amresco, Solon, OH, USA) in PBS. Then, cells were blocked for 40 min with Peroxidase-Blocking Reagent (#SM801, Dako, Glostrup, Denmark) and incubated with rabbit anti-Flag antibody (#20543, 1:200, Proteintech, Wuhan, China) at 4 °C overnight, followed by 30 min of incubation with secondary AlexaFluor^TM^ 488 antibody (#A21206, 1:200, Thermo Scientific, Carlsbad, CA, USA) and 4′,6-Diamidine-2′-phenylindole dihydrochloride (#G1012, DAPI, Servicebio, Wuhan, China).

### 4.9. Statistical Analysis

The comparison of the mean values between three groups was performed using a one-way ANOVA test in the GraphPad Prism software. The comparison of the mean values between two groups was performed using Student’s *t*-test in the GraphPad Prism software.

## 5. Conclusions

In summary, our study demonstrates the pivotal roles of exon 3 in the expression and localization of PD-L1. Exon 3 inclusion requires two key ESEs in exon 3. Two ASOs targeting these ESEs were able to promote the exon 3 skipping of PD-L1 and reduce the suppression of cancer cells on immune cells. Moreover, these two ASOs can inhibit cell growth and induce cell pyroptosis in OSCC cells.

## Data Availability

Data are freely available from the corresponding author upon reasonable request.

## Supplementary Materials

The supporting information can be downloaded at: https://www.mdpi.com/article/10.3390/ijms24098193/s1.
