# Supplementary material for: PD-L1 Exon 3 Is a Hidden Switch of Its Expression and Function in Oral Cancer Cells
Source: Int J Mol Sci. 2023 May 3;24(9):8193. doi: 10.3390/ijms24098193 (PMC10178889; doi:10.3390/ijms24098193)
Supplement: Supplementary file 1 [file ijms-24-08193-s001.zip › ijms-2264443-supplementary.pdf]

Table S1. The sequences of the antisense oligonucleotides (ASOs).

ASOs	Sequences
mt21 ASO-1	GAGAGCTGGTCCTTCAACAG
mt21 ASO-2	AGAGCTGGTCCTTCAACAGC
mt21 ASO-3	GAGCTGGTCCTTCAACAGCC
mt21 ASO-4	AGCTGGTCCTTCAACAGCCG
mt21 ASO-5	GCTGGTCCTTCAACAGCCGG
mt21 ASO-6	CTGGTCCTTCAACAGCCGGG
mt21 ASO-7	TGGTCCTTCAACAGCCGGGC
mt21 ASO-8	GGTCCTTCAACAGCCGGGCC
mt21 ASO-9	GTCCTTCAACAGCCGGGCCC
mt21 ASO-10	TCCTTCAACAGCCGGGCCCT
mt29 ASO-1	CACCATAGCTGATCATGCAG
mt29 ASO-2	ACCATAGCTGATCATGCAGC
mt29 ASO-3	CCATAGCTGATCATGCAGCG
mt29 ASO-4	CATAGCTGATCATGCAGCGG
mt29 ASO-5	ATAGCTGATCATGCAGCGGT
mt29 ASO-6	TAGCTGATCATGCAGCGGTA
mt29 ASO-7	AGCTGATCATGCAGCGGTAC
mt29 ASO-8	GCTGATCATGCAGCGGTACA
mt29 ASO-9	CTGATCATGCAGCGGTACAC
mt29 ASO-10	TGATCATGCAGCGGTACACC
mt29 ASO-11	GATCATGCAGCGGTACACCC

Table S2. The sequences of primers used for RT-PCR.

**Gene**	**Forward**	**Reverse**
PD-L1 (minigene)	CATGACCTACTGGCATTTGCTG	GCTCCTCGCCCTTGCTCACCA
PD-L1-L	ATGGTGGTGCCGACTACAAG	GGAATTGGTGGTGGTGGTCT
PD-L1-S	TTGCTGAACGCCCCATACAA	TCCAGATGACTTCGGCCTTG
PD-L1-L&S	CATGACCTACTGGCATTTGCTG	CCACTCAGGACTTGATGGTCACT
Total PD-L1	ATGCCTTGGTGTAGCACTGA	GCTGGATTACGTCTCCTCCAAA
GAPDH	GAAGGTGAAGGTCGGAGTC	GAAGATGGTGATGGGATTTC
GFP	CCGTAGGTGGCATCGCCCTC	ATGGTGAGCAAGGGCGAGGA
